# Role of CT-based body composition parameters in the course of COVID-19

**DOI:** 10.55730/1300-0144.5886

**Published:** 2024-04-24

**Authors:** Elçin AYDIN, Begüm ERGİN, Ezgi GÜVEL VERDİ, Özge COŞKUN, Şükrü ŞAHİN, Ali Haydar BAYKAN, Hilal ŞAHİN

**Affiliations:** 1 Department of Radiology, Tepecik Training and Research Hospital, Health Sciences University, İzmir, Turkiye; 2 Department of Radiology, Faculty of Medicine, Adıyaman University, Adıyaman, Turkiye

**Keywords:** COVID-19, risk factors, body composition, prognosis, thorax

## Abstract

**Background/aim:**

A significant correlation is observed between the course of coronavirus disease 2019 (COVID-19) and body composition parameters including visceral fat quantification, muscle mass, and hepatic attenuation. The aim of the study was to investigate the correlation between the extent of lung involvement and various computed tomography (CT) parameters, as well as laboratory findings in COVID-19 patients.

**Materials and methods:**

A retrospective analysis was conducted on 72 adult patients with laboratory-confirmed severe acute respiratory syndrome coronavirus 2 infection who underwent two consecutive thorax CT scans at least 2 weeks apart. The patients were divided into two groups, as progressive and nonprogressive, based on the presence of two consecutive CT scans. Skeletal muscle area (SMA), subcutaneous fat area (SFA), visceral fat area (VFA), total fat area (TFA), liver-to-spleen (L/S) density ratio, and laboratory findings were compared between the groups. The correlation between the extent of lung involvement and CT parameters, as well as the laboratory findings were assessed.

**Results:**

A total of 72 patients were included in the study, with 34 (47.2%) females and 38 (52.8%) males. Hemoglobin levels were significantly lower in the progressive group compared to the nonprogressive group. C-reactive protein (CRP) values were higher in the progressive group at follow-up. The nonprogressive group exhibited decreases in the SFA, VFA, and TFA, while liver density increased. The progressive group showed a decrease in the twelfth thoracic vertebra (T12) paravertebral muscle area and muscle index.

**Conclusion:**

In the comparison of the laboratory and radiological data in the course of COVID-19, white blood cell (WBC) and neutrophil counts increased, SMA T12, and the skeletal muscle index (SMI) decreased in the lung progressive group. Hemoglobin and CRP levels at admission may indicate disease progression. Future studies are warranted to increase the reliability with larger series.

## 1. Introduction

Severe acute respiratory syndrome coronavirus 2 (SARS-CoV-2), which causes coronavirus disease 2019 (COVID-19), has rapidly spread around the globe since December 2019 [1]. This prompted the World Health Organization (WHO) to classify it as a global pandemic on March 11th, 2020^1^

The Reverse transcriptase-polymerase chain reaction (RT-PCR) is currently the most reliable and effective way to diagnose the factor [2]. Due to its widespread availability and its role in enabling fast pneumonia diagnosis, chest computed tomography (CT) is fundamental to diagnosing and managing COVID-19 [3,4].

The infection can cause mild to moderate COVID-19 and COVID-19 pneumonia, leading to admission to the intensive care unit (ICU) and even death for some patients [5]. Thus, it is essential to be able to estimate which individuals will experience the progression of the disease and what their prognosis will be.

Studies have identified several risk factors that make individuals more susceptible to severe

illness, including age, male sex, diabetes mellitus (DM), hypertension, cardiopulmonary diseases, obesity, and sarcopenia [6–12].

In the literature, there is a wealth of academic studies on the subject of sarcopenia, obesity, and sarcopenic obesity. In recent years, there have been numerous studies investigating the relation between sarcopenic obesity and long-term illnesses, including cancer and other inflammatory diseases [13,14]. It has been observed that 10% or more of the global population have sarcopenia and sarcopenic obesity, both of which are associated with prolonged hospital stays and an elevated mortality rate. [15]. Improved thoracic muscle quality has been linked to a decreased rate of hospitalization, invasive mechanical ventilation (IMV), and death [16].

Obesity has almost become a pandemic in developed countries and is a significant risk factor for many diseases [17]. It is widely accepted that there is a direct link between obesity and respiratory diseases such as asthma, obstructive sleep apnea syndrome, and COVID-19 pneumonia [17,18].

Unenhanced CT scans have been utilized to reveal hepatic steatosis by measuring the liver-to-spleen (L/S) ratio. Therefore, the L/S ratio may be a useful tool in the analysis of liver damage as it demonstrates alterations in the attenuation of hepatic tissue [6].

Laboratory studies could be crucial in determining the severity of COVID-19 infection, distinguishing between severe and nonsevere cases. Abnormal laboratory findings among COVID-19 patients are frequently characterized by increased lactate dehydrogenase, alanine aminotransferase (ALT), aspartate aminotransferase (AST), total bilirubin (BIL), creatine kinase (CK), and creatinine (Cr) levels, and leukocytes and neutrophils counts as well as a decrease in the albumin level and lymphocyte count [19].

The aim of this study was to evaluate the relationship between clinical, laboratory, and CT-based body composition findings such as visceral fat quantification, muscle mass, and hepatic attenuation, and radiological COVID-19 lung progression. Furthermore, this is the first study investigating correlations between body composition, laboratory and clinical results, and COVID-19 pneumonia concurrently in the English literature.

## 2. Materials and methods

### 2.1. Patients and study design

Eligible patients were identified by searching the radiological information from our institutional PACS system retrospectively. From March 2019 to January 2022, among 1791 patients who were suspected of having COVID-19 pneumonia, those who had 2 successive thorax CTs at least 2 weeks apart with consecutive laboratory results were selected for inclusion. Among the total number of patients, only 72 met those criteria. The research was only conducted with participants who were 18 years of age or older. Individuals with RT-PCR tests that yielded positive results, yet who did not receive thorax CT scans were excluded. Moreover, contrast-enhanced CT scans and abundant artifacts on images that would lead to incorrect interpretation were other exclusion criteria. Patient data at hospital admission were obtained from digital records and encompassed demographics, body mass index (BMI), symptoms, comorbidities, and laboratory test results.

Two radiologists (EGV and OC) with 7 years of experience in abdominal radiology reviewed the images and patient histories collectively. The CT scans were examined initially, and subsequently, the medical histories of patients were reviewed in a blinded manner. As a result, 72 patients with RT-PCR-confirmed SARS-CoV-2 infection who underwent two consecutive thorax CT at least 2 weeks apart were retrospectively reviewed for the assessment of demographical, clinical, and laboratory data, and outcomes of the lung CT scores (nonprogressive vs. progressive) [20,21]. Authorization for this retrospective study was granted by our institutional organization and approved by the local institutional review board.

### 2.2. Image acquisition and analysis

All the CT scans were performed using a 128-slice-CT scanner (Somatom Definition AS, Siemens Healthineers; Erlangen, Germany). Each thorax CT scan was performed with the patient in the supine position, and during a single inspiratory breath hold when feasible. Axial images were obtained that ranged from the thoracic inlet to the upper abdomen, including the upper or middle poles of the kidneys. All the CT scans were performed without the administration of a contrast agent.

**Figure 1:**
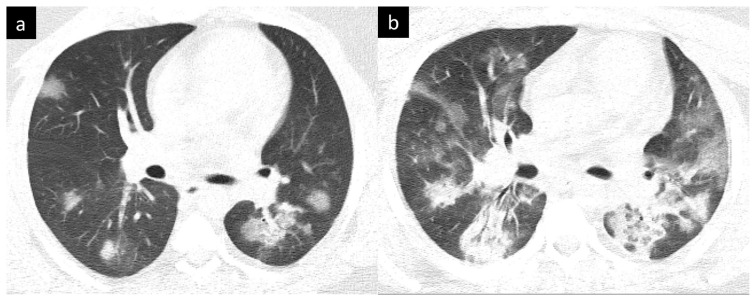
CT findings of progressive COVID-19 pneumonia. **a**. Axial CT image showing multifocal rounded focal consolidations and ground-glass opacities with predominant peripheral distribution in a 34-year-old man with a positive RT-PCR test. Initial lung CT score was 10. **b** Axial CT image showing that consolidations and ground glass opacities in the corresponding segments became more evident at follow-up. Lung CT score was scaled up to 20.

The degree of the involvement of each lung lobe was assessed, and classified as score 0, no involvement; score 1, less than 5% involvement; score 2, 5%–25% involvement; score 3, 26%–49% involvement; score 4, 50%–75% involvement; and score 5, greater than 75% involvement of the lobe. By adding together the 5 lobe scores, an overall lung total severity score was obtained. According to this scoring system, the minimum and maximum values for the lung CT scores were 0 and 25, respectively [6,20,22]. Data were compared in the progressive and nonprogressive groups based on the lung score. The suggested classification of Frankone et al. [20] for the extent of the disease was used. In compliance with this scoring system, the patients were divided into 2 groups and determined as the progressive and nonprogressive groups (Figures 1a and 1b). The determination of progressive/nonprogressive disease was assessed in consensus by two radiologists.

The progressive group comprised patients whose lung CT scores increased at follow-up, whereas nonprogressive group comprised patients with the same or decreased CT scores. From two consecutive thorax CTs, the skeletal muscle area (SMA) and density of the paravertebral muscles at the level of the fifth thoracic (T5) and T12 vertebrae (SMD), skeletal muscle index (SMI), subcutaneous fat area (SFA), visceral fat area (VFA), total fat area (TFA) from the abdominal sections, and L/S ratio and abdominal circumference were measured by two radiologists independently using the AWI server application (AWI Server, 3.2 Ext 1.0; GE Healthcare, Milwaukee, WI, USA). Measurements were performed more than once to check reproducibility, and the interobserver variability was calculated for measurement of the CT parameters.

**Figure 2:**
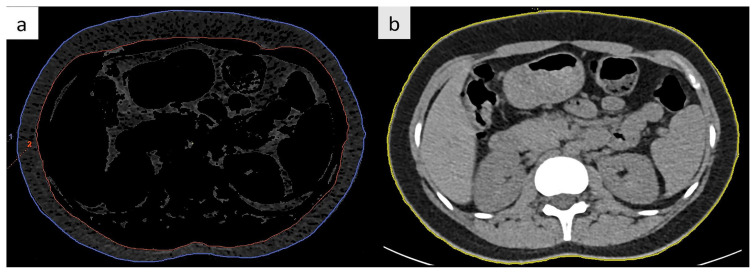
Measurements using the automated postprocessing application. **a** The area between the blue and red ROIs demonstrates subcutaneous adipose tissue. The area within the red ROI indicates visceral adipose tissue. Fat is identified as dark grey. **b** The yellow ROI shows the abdominal circumference.

Calculations of fat content and abdominal circumference were measured manually at the first slice caudal to the pleural recess (Figures 2a and 2b) [23]. The SFA was calculated by subtracting the VFA from the TFA. The fat threshold values were set between −190 and −30 Hounsfield unit (HU) [15].

**Figure 3:**
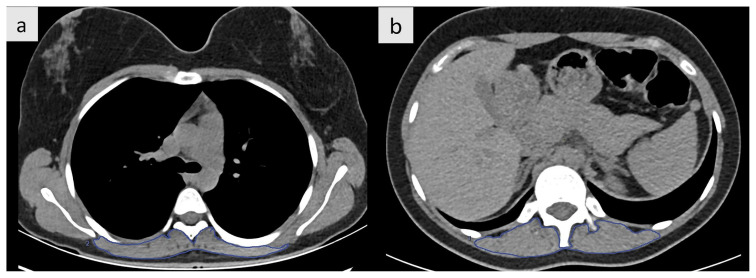
Example of paravertebral muscle delineation (blue outlining). **a** At the T5 vertebra and **b** at the T12 vertebra.

In order to measure the muscle size, an area measurement of the paravertebral muscles at the T5 and T12 vertebrae was performed (Figures 3a and 3b). Muscle outlines were manually contoured. A muscle-specific threshold was then used to determine the SMA. The SMI was calculated by taking the SMA (cm^2^) and dividing it by the square of the body height (m^2^). The threshold values for muscle were set between −29 and +150 HU [15].

As not all the patients had recorded height and weight measurements, so the vertebral size was used as a representation of the BMI to assess the SMI. This was done based on the study of Waduud et al., who demonstrated how to convert vertebral body parameters into estimated height [24].

**Figure 4:**
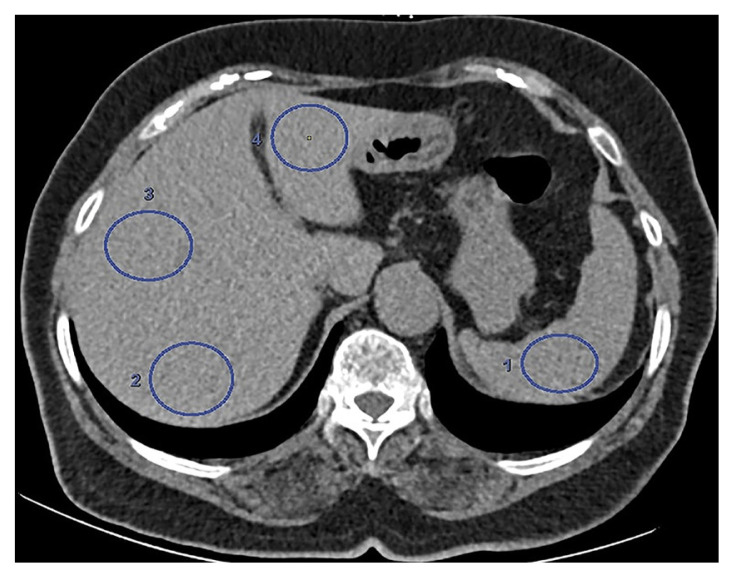
A single ROI was placed in the left liver lobe (4) to calculate hepatic attenuation values, while 2 ROIs were placed in the right liver lobe (2 and 3). Splenic attenuation was obtained by placing 1 ROI (1).

The calculation of the hepatic attenuation values was done by placing 2 regions of interest (ROIs) with a size of at least 100 mm^2^ in the right lobe of the liver anteroposteriorly, as well as one ROI in the left lobe with attention to avoiding major vascular structures (Figure 4). Splenic attenuation was obtained by placing one ROI, greater than 100 mm^2^ in area. The L/S ratio was obtained by taking the mean of the HU measurements of both liver lobes and dividing it by the HU of the spleen. Careful consideration was taken to place the ROIs in exactly the same location in both CT scans.

### 2.3. Statistical analysis

The Kolmogorov–Smirnov test was used to determine if the data conformed to normal distribution. Categorical variables were expressed as percentages, and content showing nonparametric variables were expressed as the median and range. The chi-squared test was used to compare the qualitative data. Dependent numerical data showing nonparametric distribution were evaluated using the Wilcoxon test. Spearman’s rho analysis was performed to evaluate the relationship between the extent of lung involvement and laboratory findings, and the body composition parameters. The intraclass correlation coefficient (ICC) was used for measurement of the CT parameters between two observers. Spearman’s rho values between ±0.2 and ±0.4, ±0.4 and ±0.6, ±0.6 and ±0.8, and ±0.8 and ±1 were considered as weak, moderate, strong, and very strong association, respectively. p < 0.05 was considered statistically significant for all the tests.

## 3. Results

### 3.1. Demographic data and clinical features

Of the 72 patients included in the study, 34 (47.2%) were female and 38 (52.8%) were male. The mean age was 59.94 ± 16.57 (range: 25–88) years. The mean BMI was 23.83 kg/m^2^ ± 1.87 for the males and 24.09 kg/m^2^ ± 3.13 for the females (p = 0.67). Of the 8 (11.1%) patients requiring admission to the ICU, 3 were female and 5 were male. The mean length of stay in the ICU was 11.96 days: 11.06 days for the females and 12.76 days for the males (p = 0.531). The minimum length of stay was 2 days, and the maximum was 35 days. During the study period, 3 (4.1%) patients, 1 female and 2 males, died, and 69 (95.9%) patients were discharged. The most common comorbidities were hypertension (n = 27, 37.5%), DM (n = 19, 26.4%), coronary artery disease (CAD) (n = 8, 11.1%), chronic obstructive pulmonary disease (COPD) (n = 5, 6.9%), chronic renal failure (CRF) (n = 5, %) 6.9), malignancy (n = 4, 5.6%). Of these, 11 (15.3%) had more than one comorbidity (Table 1). The nonprogressive group consisted of 44 (61.1%) patients and the progressive group consisted of 28 (38.9%). Patients in the progressive group had a longer hospital stay (p = 0.017). While three (5%) patients died in the progressive group, none of the patients died in the nonprogressive group.

### 3.2. Laboratory findings

Hemoglobin levels were significantly lower in the progressive group than in the nonprogressive group at admission. Hemoglobin levels were significantly lower in the progressive group than in the nonprogressive group at both admission and follow-up (Table 2).

At hospital admission, the direct BIL concentration was significantly higher (p = 0.017) and the Cr level was significantly lower (p = 0.04) in the nonprogressive group compared to the progressive group. At follow-up, the C-reactive protein (CRP) values were significantly higher (p = 0.044) in the progressive group compared to the nonprogressive group. The in-group comparison revealed a significant increase in the platelet (p < 0.001) and ALT (p = 0.013) levels from hospital admission to follow-up only in the nonprogressive group. Likewise, the WBC and neutrophil counts increased significantly (p = 0.006, p = 0.015, respectively) only in the progressive group.

### 3.3. CT parameters

Interobserver agreement was almost perfect between the readers for measurement of CT parameters (ICC range: 0.849–0.970), except for the L/S density ratio with substantial agreement (ICC: 0.735) (Table 3).

There was no significant difference between the progressive and nonprogressive groups in any CT parameters at admission (Table 4). Moreover, no significant differences were found in any of the CT parameters between the 2 groups at follow-up. In the nonprogressive group, the SFA, VFA, and TFA decreased significantly from hospital admission to follow-up (p = 0.009, p = 0.009, and p = 0.002, respectively), whereas the liver density increased significantly (p = 0.02). In the progressive group, the T12 paravertebral muscle area and muscle index decreased in the follow-up CT.

At admission, a weak positive correlation was observed between the extent of lung involvement and the white blood cell (WBC) count direct BIL level. In addition, a weak negative correlation was seen between lung involvement and liver density and the L/S density ratio. Moreover, a moderate positive correlation was found between lung involvement and the CRP levels (Table 5). Moreover, there was a weak negative correlation between lung involvement and liver density (p = 0.037). Furthermore, at follow-up, the correlation between lung involvement and the neutrophil count, procalcitonin and Cr levels were weakly positive, whereas there was a moderate positive correlation with the CRP levels.

## 4. Discussion

This study aimed to evaluate the association between clinical, laboratory, and CT-based body composition assessments, including visceral fat estimation, muscle mass, hepatic attenuation, abdominal circumference, and the radiologic course of COVID-19 pneumonia. The body composition measures were obtained by routinely performed thorax CT scans on COVID-19 patients.

Various studies have shown that obese patients are more likely to experience severe COVID-19 symptoms with increased morbidity, such as an increased rate of hospitalization and the need for IMV [25]. It is well-known that different types of adipose tissue depots cause obesity. This includes visceral adipose tissue and subcutaneous adipose tissue, which bring forth distinct degrees of hazard for metabolic disorders and cardiovascular risks [26]. Petersen et al. [8] reported that visceral adipose tissue and the CT-derived upper abdominal circumference are significant indicators of severe courses of COVID-19. In the current study, no correlation was found between the adipose tissue composition and disease progression. This could be attributed to the fact that the CT scans conducted at follow-up were ordered at short time intervals. Moreover, it is possible that the body composition parameters measured from other body levels were not as reliable as those taken from abdominal CT scans at the third lumbar (L3) vertebra. Additionally, it was noted that the VFA diminished in the progressive group at follow-up. This could potentially be linked to inadequate nutrition during a severe illness.

Visceral fat accumulation can have a significant impact on the prognosis of diseases. Results from research have suggested that nonalcoholic fatty liver disease can cause an elevated mortality rate in individuals who have community-acquired pneumonia [27]. The hypothesis in the present study was that hepatic steatosis and a decrease in the L/S ratio could worsen COVID-19 outcomes, considering its effects on human metabolism and its correlation with obesity. In a study by Guler et al. [6], a decrease in the L/S ratio was observed in COVID-19 patients with elevated lung CT scores. In the current study, there was a weak correlation between the liver density, L/S density ratio, and disease progression. It is possible that the positioning of the upper arms along the sides of some patients may have produced arm-related noise that could have influenced the attenuation values in some cases.

Cross-sectional images can be used to measure skeletal muscle mass and the SMI. A range of disorders such as COVID-19 can be impacted by these parameters, which have predictive and prognostic implications. The SMI is used to consider the effect of body height on muscle tissue, which is calculated by dividing the SMA by the square of the body height. The SMI can be considered as a more standardized parameter [12]. Another hypothesis in this study was that sarcopenia and sarcopenic obesity have a considerable and detrimental effect on the progression of the disease. In the current study, no relation was found between the muscle measurements at admission and progression of the disease. In our opinion, a lack of proper nutrition during a serious illness in combination with the inflammatory changes caused by COVID-19 might have caused decrease in the SMA and SMI T12 at follow-up.

Multiple laboratory have been proposed to predict mortality in those with COVID-19 based on clinical parameters [19,28]. One study demonstrated that increased neutrophil, CRP, procalcitonin, AST, ALT, and total BIL values and decreased lymphocytes, platelets, and albumin values have a prognostic value for COVID-19 [19]. In the present study, neutrophil counts increased significantly in the progressive group at follow-up (p = 0.006 and p = 0.015, respectively). Moreover, the CRP levels were higher at follow-up in the progressive group. The increase in the serum direct BIL concentration was significant (p = 0.017). There was a weak positive correlation between the extent of lung involvement and the WBC, direct BIL levels, procalcitonin, and Cr levels. A moderate positive correlation between the CRP levels also was found.

This study had several limitations. First, it was retrospective, conducted at a single center, and had small number of patients. Second, no baseline images of the patients were taken into consideration, and any that were present were not utilized. Therefore, some overlapping features of COVID-19 pneumonia and other diseases may have caused a misinterpretation of the images as progression. Third, a single slice of adipose tissue was measured in the area where the lung parenchyma was not visible caudal to the pleural recess. Hence, the level was not validated such as for those obtained at the L3. Forth, from a technical standpoint, the positioning of the upper arms along the sides of the patients may have caused arm-related noise that could have impacted the attenuation values. In some cases, this may have led to inaccurate measurement of the density of the muscle and fat tissue. Additionally, not all the patients had recorded heights. As a result, the anteroposterior diameter of the T12 vertebra was used to assess the height of some of the patients, a method that was previously validated [24]. Furthermore, in our hospital, patients with quick health deterioration and transport problems are monitored clinically and with traditional chest X-ray techniques due to their convenient mobility. Thus, a considerable number of patients were excluded from this study.

In conclusion, thorax CT scan plays a crucial role in detecting COVID-19. Thorax CT can be used to measure body composition parameters such as the SMA and T12 SMI, and these parameters seem to decrease as the severity of COVID-19 pneumonia increases. Additionally, a low level of hemoglobin and increased CRP levels at admission may predict progression of the disease. Moreover, WBC and neutrophil counts, CRP, and procalcitonin values obtained at follow-up may be associated with progression of the disease.

## Figures and Tables

**Table 1:** Demographic data and clinical features of the 2 COVID-19 patient groups.

Parameters	Nonprogressive group (n = 44)	Progressive group (n = 28)	p-value
Age	62 (25–88)	63.50 (30–86)	0.632
Sex			0.706
Male	24 (54.5%)	14 (50%)	
Female	20 (45.5%)	14 (50%)	
Comorbidities	23 (52.3%)	15 (53.4%)	0.435
Hypertension	18 (40.9%)	9 (32.1%)	
DM	10 (22.7%)	9 (32.1%)	
CAD	3 (6.8%)	7 (25%)	
Malignancy	3 (6.8%)	1 (3.6%)	
CRF	3 (6.8%)	2 (7.2%)	
COPD	3 (6.8%)	2 (7.2%)	
Treatment			0.993
Favipiravir	30 (68.2%)	19 (67.9%)	
Hydroxychloroquine	9 (20.4%)	6 (21.4%)	
Oseltamivir	5 (11.4%)	3 (10.7%)	
Length of hospital stay (days)	7 (2–35)	12 (2–75)	0.017
ICU admission	2 (2.8%)	6 (8.3%)	0.049
Deceased	0 (0%)	3 (4.1%)	

Data are given as the n (%) or median (minimum–maximum).

**Table 2:** Comparison of the laboratory findings in the COVID-19 progressive and nonprogressive groups.

Parameter	Nonprogressive group (n = 44)		Progressive group (n = 28)		Between group comparison (p)		Hospital admission and follow-up comparison (p)	
	At hospital admission Median (min–max)	At follow-up Median (min–max)	At hospital admission Median (min–max)	At follow-up Median (min–max)	At hospital admission^a^	At follow-up^b^	Nonprogressive group^c^ (p-value)	Progressive group^d^
WBC	5.55 (3.10–30.40)	6.70 (1.7–21.9)	5.2 (1.60–13.4)	7.45 (2.10–23.00)	0.79	0.822	0.064	**0.006**
Neutrophil	3.7 (1.4–21.80)	4.7 (1.2–16.7)	3.75 (0.8–11.0)	5.45 (1.0–21.50)	0.876	0.552	0.052	**0.015**
Lymphocyte	1.0 (0.3–26.50)	1.45 (0.20–3.10)	1.0 (0.40–2.0)	1.10 (0.4–2.90)	0.862	0.619	0.169	0.099
Hb	13.4 (10.3–15.70)	13.4 (6.5–15.5)	12.8 (7.3–15.5)	12.25 (7.40–16.00)	**0.03**	**0.025**	0.101	0.07
Platelet	193 (83–374)	232 (34–472)	188 (117–384)	188 (102–571)	0.808	0.296	**<0.001**	0.072
CRP	37.5 (1–327)	24.7 (1.0–200)	31.5 (2–296)	57 (1–226)	0.808	**0.044**	0.131	0.094
Procalcitonin	0.04 (0.01–6.06)	0.06 (0.01–0.73)	0.045 (0.01–1.04)	0.07 (0.01–5.71)	0.744	0.636	0.245	0.247
D-dimer	1000 (190–10,410)	840 (190–12,150)	830 (220–7920)	1285 (250–6610)	0.862	0.236	0.250	0.124
AST	32 (12–503)	30 (0–489)	27 (15–56)	30 (15–168)	0.229	0.853	0.937	0.101
ALT	27 (6–337)	37 (1–651)	19 (7–67)	23 (7–221)	0.080	0.160	**0.013** c	0.037
Total BIL	0.63 (0.3–3.08)	0.64 (0.07–1.9)	0.5 (0.21–1.25)	0.5 (0.06–1.08)	0.139	0.062	0.316	0.456
Direct BIL	0.16 (0.05–0.49)	0.15 (0.03–0.57)	0.11 (0.05–0.35)	0.11 (0.04–0.34)	**0.017**	0.088	0.638	0.885
Cr	1.0 (0.5–11.30)	1.02 (0.6–8.90)	1.2 (0.8–8)	1.0 (0.60–3.20)	**0.04**	0.401	0.801	0.898

Bold font indicates significance at p < 0.05, data are given as the median (minimum–maximum). WBC: white blood cells (4.5–11 × 10^3^/μL), neutrophil count (2.02–7.46 × 10^3^/μL), lymphocyte count (1–3.38 × 10^3^/μL), Hb: hemoglobin (11.7–16 g/dL), platelet count (150–450 × 10^3^/μL), CRP: C-reactive protein (0–5 mg/L), procalcitonin (0.04–0.1 μg/L), d-dimer (<550 μg/L FEU), AST: aspartate amino transferase (normal limits, <35 U/L), ALT: alanine aminotransferase (normal limits, <45 U/L), BIL: total bilirubin (0.1–1 mg/dL), direct BIL (mg/dL), and Cr: creatinine (0.6–1.1 mg/dL).

a CT parameter comparison of the progressive and nonprogressive groups at hospital admission.

b CT parameter comparison of the progressive and nonprogressive groups at follow-up.

c Nonprogressive group CT parameter comparison at hospital admission and follow-up.

d Progressive group CT parameter comparison at hospital admission and follow-up.

**Table 3:** Evaluation of the interrater agreement using intraclass correlation.

	ICC coefficient	95% CI	p-value
SMI T5	0.858	0.773–0.911	<0.001
SMI T12	0.840	0.745–0.900	<0.001
AC (cm)	0.970	0.953–0.982	<0.001
SFA (cm^2^)	0.945	0.912–0.966	<0.001
VFA (cm^2^)	0.914	0.862–0.946	<0.001
TFA (cm^2^)	0.920	0.872–0.950	<0.001
L/S density	0.735	0.577–0.834	<0.001
Liver HU	0.875	0.801–0.922	<0.001

ICC: Intraclass correlation coefficient, CI: confidence interval.

**Table 4:** CT parameter comparison of the COVID-19 progressive and nonprogressive groups.

Parameter	Nonprogressive group (n = 44)		Progressive group (n = 28)		Between group comparison (p)		Hospital admission and follow-up comparison (p-value)	
	At hospital admission Median (min–max)	Follow-up Median (min–max)	At hospital admission Median (min–max)	Follow-up Median (min–max)	At hospital admission^a^	Follow-up^b^	Nonprogressive group^c^ (p)	Progressive group^d^
SMA T5 (cm^2^)	11.31 (6.28–18.92)	10.73 (4.34–21.71)	10.81 (5.96–19.58)	10.87 (6.15–23.59)	0.786	0.738	0.434	0.699
SMI T5	4.00 (2.21–7.31)	3.92 (1.66–7.49)	3.81 (1.99–7.06)	3.95 (1.99–7.90)	0.652	0.632	0.424	0.657
SMA T12 (cm^2^)	30.05 (14.44–49.35)	27.35 (13.45–55.63)	31.97 (17.29–52.48)	29.49 (15.03–36.48)	0.619	0.862	0.059	**0.005**
SMI T12	10.47 (5.37–17.14)	9.76 (4.45–21.87)	11.38 (5.86–17.63)	10.33 (4.92–13.96)	0.389	0.954	0.137	**0.004**
AC (cm)	103.95 (73.07–124.93)	102.68 (72.16–123.05)	105.77 (93.44–124.01)	106.25 (94.33–115.58)	0.579	0.556	0.077	0.124
SFA (cm^2^)	114.56 (8.49–361.56)	109.59 (12.11–347.45)	128.98 (7.28–335.68)	128.00 (8.99–351.78)	0.278	0.216	**0.009**	0.820
VFA (cm^2^)	140.166 (12.61—471.15)	137.41 (7.63—328.73)	160.32 (16.94—338.30)	148.03 (17.63—345.83)	0.862	0.556	**0.009**	0.855
TFA (cm^2^)	275.38 (21.11–642.86)	270.48 (19.75–617.52)	304.88 (24.22–488.19)	288.43 (26.84–520.17)	0.611	0.230	**0.002**	0.964
L/S density	1.0 (0.30–1.66)	1.08 (0.35–2.21)	1.03 (0.54–2.26)	1.05 (0.50–1.53)	0.808	0.773	0.061	0.982
Liver HU	58.20 (15.55–74.97)	59.15 (18.55–72.55)	55 (31.75–74.66)	55.53 (21.70–69.30)	0.899	0.053	**0.023**	0.227
Spleen HU	55.15 (36.90–77.10)	54.90 (27.80–70.70)	53.30 (31.20–68.50)	52.50 (12.60–67.40)	0.278	0.178	0.829	0.855

Bold font indicates significance at p < 0.05, data are given as the median (minimum–maximum). SMA: Skeletal muscle area, T5: fifth thoracic vertebra, T12: twelfth thoracic vertebra, AC: abdominal circumference, SMI: skeletal muscle index.

a CT parameter comparison of the progressive and nonprogressive groups at hospital admission.

b CT parameter comparison of the progressive and nonprogressive groups at follow-up.

c Nonprogressive group CT parameter comparison at hospital admission and follow-up.

d Progressive group CT parameter comparison at hospital admission and follow-up.

**Table 5:** Relationship between the laboratory and CT parameters and extent of lung involvement at hospital admission and follow-up.

Variables	Extent of lung involvement at hospital admission		Extent of lung involvement at follow-up	
	Spearman’s rho coefficient	p-value	Spearman’s rho coefficient	p-value
Age	0.164	0.168	0.090	0.452
WBC	**0.241**	**0.042**	0.230	0.051
Neutrophil	0.159	0.182	**0.300**	**0.011**
Lymphocyte	0.175	0.141	−0.187	0.116
Hb	−0.117	0.329	−0.293	0.013
Platelet	0.056	0.639	0.062	0.606
CRP	**0.548**	**<0.001**	**0.460**	**<0.001**
Procalcitonin	0.157	0.189	**0.279**	**0.018**
D-dimer	0.346	**0.003**	0.185	0.126
AST	0.104	0.384	−0.004	0.971
ALT	0.053	0.658	−0.057	0.635
Total BIL	0.108	0.367	0.004	0.972
Direct BIL	**0.297**	**0.011**	0.014	0.904
Cr	0.159	0.183	**0.288**	**0.014**
SMA T5 (cm^2^)	−0.132	0.269	−0.068	0.569
SMI T5	−0.122	0.306	−0.045	0.705
SMA T12 (cm^2^)	−0.055	0.646	−0.014	0.910
SMI T12	−0.108	0.365	−0.004	0.976
AC (cm)	0.384	0.104	0.154	0.196
VFA	0.598	0.063	0.012	0.923
SFA	0.015	0.898	0.151	0.204
TFA	0.051	0.673	0.113	0.344
Fat density	0.130	0.277	−0.117	0.329
L/S density	**−0.271**	**0.021**	−0.150	0.207
Liver density	**−0.323**	**0.006**	**−0.246**	**0.037**
Spleen density	−0.024	0.839	0.034	0.778

Bold font indicates significance at p < 0.05.

## Data Availability

The datasets used and/or analyzed during the current study are available from the corresponding author on reasonable request.

## Footnotes

1 World Health Organization (2023). WHO Coronavirus (COVID-19) Dashboard [online]. Website https://covid19.who.int [accessed 12.12.2023].
